# Membrane Topology and Structural Insights into the Peptide Pheromone Receptor ComD, A Quorum-Sensing Histidine Protein Kinase of *Streptococcus mutans*

**DOI:** 10.1038/srep26502

**Published:** 2016-05-20

**Authors:** Gaofeng Dong, Xiao-Lin Tian, Kayla Cyr, Tianlei Liu, William Lin, Geoffrey Tziolas, Yung-Hua Li

**Affiliations:** 1 Department of Applied Oral Sciences 5981 University Ave, Halifax, NS, B3H 1W2, Canada; 2 Department of Microbiology and Immunology, 5850 College Street, Halifax, NS, B3H 4R2, Canada

## Abstract

Quorum sensing activation by signal pheromone (CSP) in *Streptococcus mutans* depends on the membrane-associated receptor ComD, which senses the signal and triggers the signaling cascade for bacteriocin production and other cell density-dependent activities. However, the mechanism of the signal recognition via the ComD receptor in this species is nearly unexplored. Here, we show that the membrane domain of the ComD protein forms six transmembrane segments with three extracellular loops, loopA, loopB and loopC. By structural and functional analyses of these extracellular loops, we demonstrate that both loopC and loopB are required for CSP recognition, while loopA plays little role in CSP detection. A deletion or substitution mutation of four residues NVIP in loopC abolishes CSP recognition for quorum sensing activities. We conclude that both loopC and loopB are required for forming the receptor and residues NVIP of loopC are essential for CSP recognition and quorum sensing activation in *S. mutans*.

Two-component signal transduction systems (TCSTSs) are the most prevalent form of signal transduction mechanisms in bacteria^1^. A typical TCSTS consists of a membrane-associated, histidine protein kinase (HPK), which senses a specific stimulus, and a cytoplasmic response regulator (RR), which enables the cell to respond to the stimulus, via regulation of gene expression^2^. The completed genomes show that each bacterial genome contains a dozen or several dozen of TCSTSs, which play important roles in signal transduction for various cellular processes, stress adaptation and virulence^3^,^4^. Many TCSTSs have been found to function as global regulators by initiating signaling cascades, in which large sets of genes can be switched on or off 5. These systems provide the major means by which bacteria communicate with each other and the outside world^6^. Although many TCSTSs are identified to regulate diverse physiological activities and virulence in bacteria^7^, relatively little is known of how a given TCSTS recognizes and transduces an extracellular signal to initiate the signaling cascade for gene expression. In many cases, signals sensed by TCSTSs are chemically undefined, so that signal recognition mechanisms of many TCSTSs remain poorly understood. Among many TCSTS systems, quorum-sensing signal pheromones are the best-studied signal molecules that can be specifically sensed by their cognate TCSTSs^8^,^9^,^10^,^11^,^12^. These TCSTS signal transduction systems may provide an excellent opportunity to study signal molecule-HPK receptor interactions in prokaryotic organisms.

*Streptococcus mutans*, a leading cariogenic pathogen that can cause dental caries worldwide, has evolved a well conserved, signaling peptide-mediated quorum-sensing system, the ComCDE^13^,^14^. Quorum sensing activation through this signaling system depends on several gene products (Fig. 1). The *comC* encodes a signal peptide precursor, which is cleaved and exported to release a 21-residue peptide through a peptide-specific ABC transporter encoded by *cslAB*^15^,^16^. The 21-residue peptide is further modified by an extracellular protease SepM to remove the *C*-terminal 3 residues and generate an 18-residue functional peptide or competence-stimulating peptide (CSP)^17^. The *comDE* encode a two-component transduction system that specifically senses and responds to CSP. When it reaches a critical concentration, CSP interacts with the ComD histidine kinase receptor of the neighboring cells and activates its cognate response regulator, ComE, via autophospharylation. The phospharylated ComE in turn activates numerous downstream genes, triggering the signaling cascade to regulate bacteriocin production^18^, genetic competence^14^, biofilm formation^9^ and stress response^19^,^20^, which are all considered as the key virulence factors in the *S. mutans* pathogenesis. The quorum sensing circuit in *S. mutans* is the system in which the signal molecule is well studied in chemical details^16^,^17^,^21^. However, relatively little is known of the membrane-spanning receptor protein ComD and its interaction with the signal molecule.

Most known peptide pheromone receptors, with the exceptions of SpaK, ComP and NisK, fall into the HPK_10_ subfamily, which includes AgrC from *Staphylococcus*^22^,^23^, ComD from *Streptococcus*^10^ and PlnB from *Lactobaccillus*^24^. All members of the HPK_10_ subfamily belong to the orthodox histidine kinases that consist of a *N*-terminal transmembrane region representing the sensor domain and *C*-terminal transmitter domain containing the conserved histidine residues in the cytoplasm^3^,^10^,^25^. It has been predicted that membrane-associated regions in the HPK_10_ subfamily consist usually of 5–8 transmembrane segments (TMSs), which are contrast to the majority of prokaryotic HPKs with a *N*-terminal membrane domain consisting of two transmembrane segments flanking an extracellular loop^3^,^10^. By *in silico* analysis of ComD proteins from *S. mutans* strains, we predicted that the membrane-associated region of the ComD protein in this species likely forms six TMSs and three extracellular loops. We hypothesized that the extracellular loops of the ComD protein might act as the CSP receptor essential of signal recognition and quorum sensing activation. To test this hypothesis, we began to investigate the membrane topology of the ComD histidine kinase receptor protein. We then examined the effects of deletion or point mutations of the extracellular loops on signal recognition and quorum sensing activation in *S. mutans*.

## Results

### The ComD membrane topology

**Figure 2:**
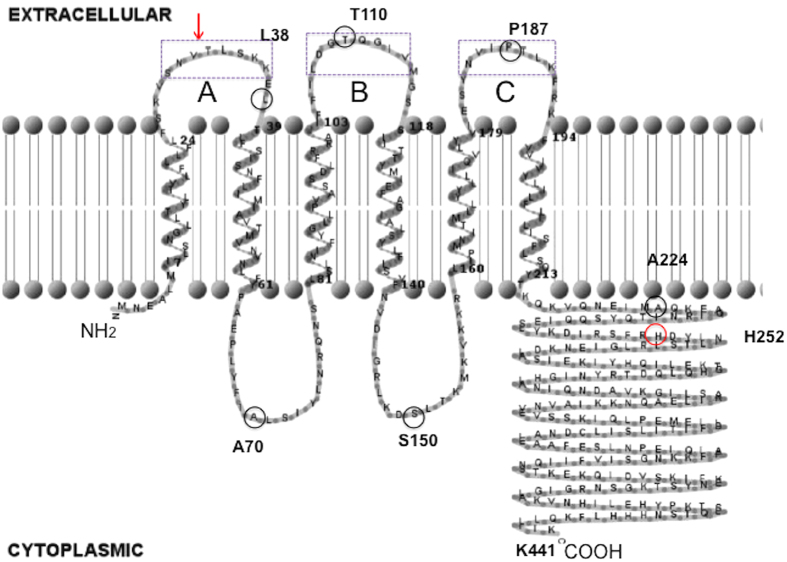
A hypothetical topology model of the ComD receptor protein in *S. mutans*. The membrane-spanning domain of ComD protein from *S. mutans* UA159 is predicted to form six transmembrane segments (TMSs) with three extracellular loops, loopA, loopB and loopC, and two intracellular loops. An arrow indicates a potential cleavable side in loopA. Small open circles indicate insertion locations by a dual *phoA*-*lacZ* fusion reporter in frame after selected codons corresponding to the amino acid residues L38, A70, T110, S150, P187 and A224. Open rectangles indicate the amino acid residues of loopA, loopB and loopC involved in the construction of in-frame deletion or substitution mutants. The conserved histidine residue (H252) in the *C*-terminal domain of the ComD protein inside the cytoplasm is also indicated.

**Figure 3:**
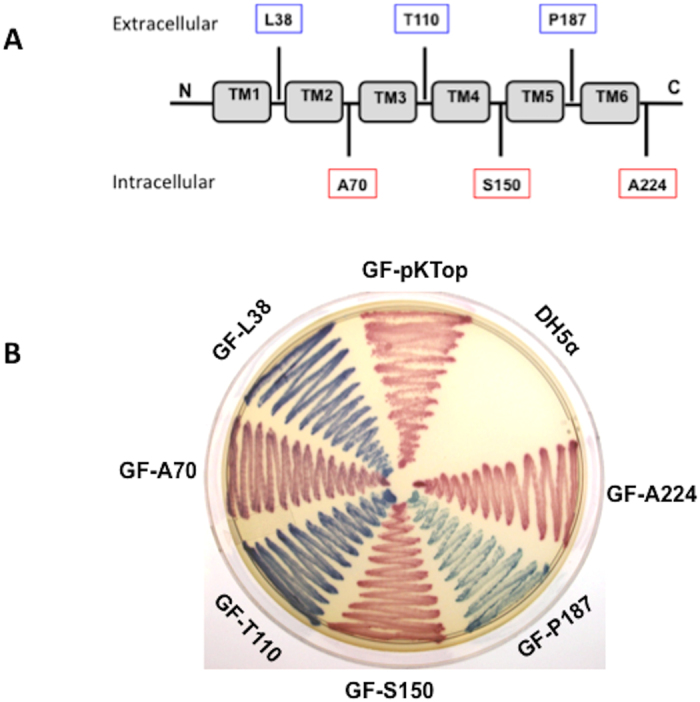
Experimental determination of the ComD membrane topology. (**A**) A schematic diagram indicates the reporter fusion points at L38 (pKTop-ComD_1-L38_), A70 (pKTop-ComD_1–A70_), T110 (pKTop-ComD_1–T110_), S150 (pKTop-ComD_1–S150_), P187 (pKTop-comD_1–P187_) and A224 (pKTop-ComD_1–A225_) of the ComD membrane-spanning region. Blue boxes indicate extracellular locations, while pink boxes indicate cytosolic locations as predicted by the hypothetical model. (**B**) The strains expressing the ComD_L38_-Pho/Lac (GF-L38), ComD_T110_-Pho/Lac (GF-T110) and ComD_P187_-Pho/Lac (GF-P187) exhibit the higher levels of phosphatase activity (blue color), indicating the extracellular location of the reporter fusion points. The strains expressing the ComD_A70_-Pho/Lac (GF-A70), ComD_S150_-Pho/Lac (GF-S150) and ComD_A225_-Pho/Lac (GF-A224) exhibit the higher levels of β-galactosidase activity (pink color), indicating the cytosolic location of the reporter fusion points. *E. coli* DH5α without pKTop (negative control) shows no color, while *E. coli* DH5α with pKTop or GF-pKTop (positive control) also shows pink color.

The ComD histidine protein kinase (HPK) of *S. mutans* is a membrane-associated protein consisting of 441 amino acid residues with a predicted molecular mass of 50.5 kDa and a pI value of 10.2^13^. The sequence alignments indicate that ComD proteins from the fifteen genome-sequence completed *S. mutans* strains are highly conserved with 96.8–100% of identity^3^,^26^,^27^. However, ComD protein of *S. mutans* only shares 22% identity and 44% similarity with those of *S. pneumoniae* strains^10^. As the first step, we obtained a hypothetical topology model of ComD protein from *S. mutans* UA159 by combining several topology prediction methods, including SOSUI (http://bp.nuap.nagoya-u.ac.jp/sosui/), SMART (http://smart.embl-heidelberg.de/smart/), TMHMM (http://www.cbs.dtu.dk/services/TMHMM-2.0/) and PSIPRED (http://bioinf.cs.ucl.ac.uk/psipred/). Based the data from these methods, a hypothetical model of the ComD topology from *S. mutans* UA159 is presented in Fig. 2. As predicted by the topology model, the ComD protein consists of two hydropathically distinct regions, the *N*-terminal membrane-spanning region (1–220 residues) and the *C*-terminal hydrophilic region (221–441 residues) inside the cytoplasm. The membrane-spanning region of the ComD protein is predicted to form six transmembrane segments (TMSs) with three extracellular loops, designated as loopA, loopB and loopC, and two intracellular loops. Based on known peptide pheromone receptors in several species of *Streptococcus*^10^, we hypothesize that these extracellular loops likely form the receptor and contribute to CSP recognition, while the *C*-terminal region inside the cytoplasm is likely responsible for the signal transduction. To validate this hypothetical model, we constructed six *comD*-*phoA-lacZ* dual fusion reporters, which represented six in-frame insertion sites (L38, A70, T110, S150, P187, A224) of the membrane-spanning region of ComD protein (Fig. 2). The resulting fusion plasmids were transformed into an *E. coli* DH5α host, generating six *comD*-*phoA-lacZ* fusion reporter strains (Fig. 3A). These fusion strains along with two control strains were used for experimental determination of the ComD membrane topology.

We then examined these fusion strains for the reporter activities by growing them on LB agar plates containing dual indicators of a blue chromogenic substrate (X-Phos) for phosphatase activity (PhoA) and a red chromogenic substrate (Salman-Gal) for β-galactosidase activity (LacZ) using the methods as described previously^28^,^29^. The results showed that the strains expressing the ComD_L38_-Pho/Lac (L38), the ComD_T110_-Pho/Lac (T110) and the ComD_P187_-Pho/Lac (P187) exhibited the higher levels of phosphatase activities (blue color), indicating the periplasmic or extracellular location of these fusion points (Fig. 3B). In contrast, the strains expressing the ComD_A70_-Pho/Lac (A70), the ComD_S150_-Pho/Lac (S150) and the ComD_A225_-Pho/Lac (A224) exhibited the higher levels of β-galactosidase activity (pink color), indicating the cytosolic location of these fusion points. In control groups, *E. coli* DH5α without pKTop (plasmid negative control) showed no color, while *E. coli* DH5α with pKTop (plasmid positive control) showed pink (β-galactosidase activity). The results from the dual *phoA-lacZ* reporter assays clearly confirm the predicted membrane topology of the ComD protein, suggesting that three extracellular loops of the *N*-terminal membrane domain of ComD protein likely form the receptor for CSP recognition in *S. mutans.*

### Confirmation of bacterial strains for investigating the extracellular loops *in vivo*

**Figure 4:**
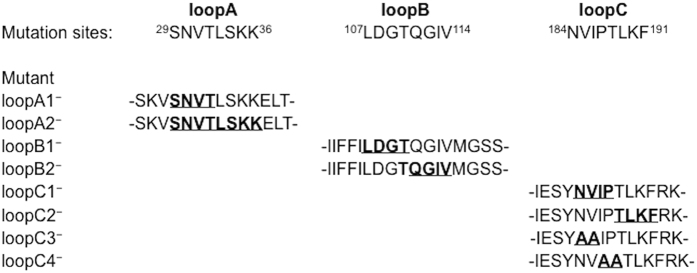
A schematic representation of the precise amino acid residues involved in the construction of extracellular loopA, loopB and loopC mutants. Only amino acid residues that constitute the extracellular loopA, loopB and loopC are shown. The bold and underlined residues indicate an in-frame deletion of each mutant. The residues AA in loopC3 and loop4 mutants indicate alanine substitution mutations.

**Figure 5:**
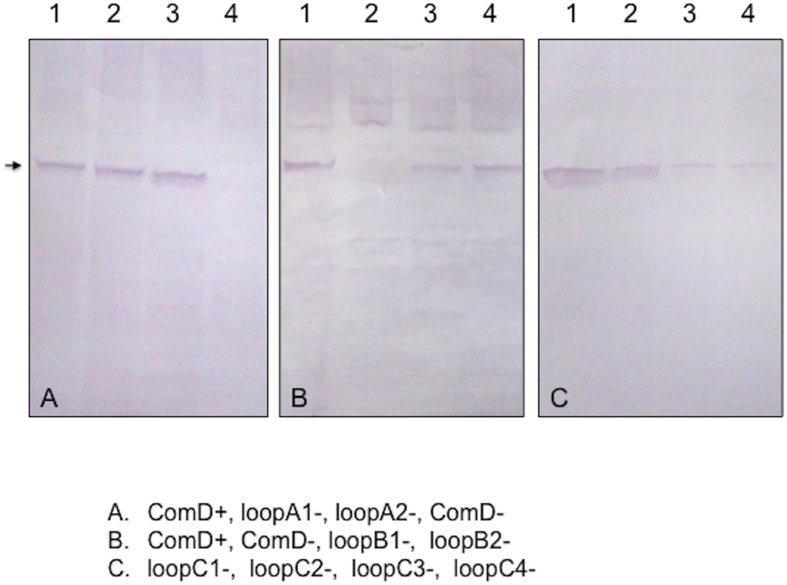
Western blot analysis of the membrane fractions of the *S. mutans* strains that constitutively express a His-tagged mutant loopA, loopB or loopC protein. All the strains were grown in THYE medium with addition of CSP to prepare the membrane proteins, which were then resolved on 10% SDS-PAGE gels and transferred onto PVDF membranes for Western blotting using the anti-His antibody. (**A**) Lanes 1–4, XT-D-H (ComD^+^), XT-A1H (loopA1^−^), XT-A2H (loopA2^−^) and XT-Pldh-H (ComD^−^); (**B**) Lanes 1–4, XT-D-H (ComD^+^), XT-Pldh-H (ComD^−^), XT-B1H (loopB1^−^) and XT-B2H (loopB2^−^); and (**C**) Lanes 1–4, XT-C1H (loopC1^−^), XT-C2H (loopC2^−^), XT-C3H (loopC3^−^) and XT-C4H (loopC4^−^). Arrow indicates 51-kDa proteins detected by Western blotting.

Having established the membrane topology of the ComD protein, we were particularly interested in the hypothesis that the ComD extracellular loops, loopA, loopB and loopC, might act as the receptor for CSP recognition and quorum-sensing activation. To test this hypothesis, we constructed three sets of *S. mutans* strains that allowed us to investigate the effects of individual extracellular loops on CSP recognition and quorum sensing activation. The first set of the strains included six in-frame deletion mutants and two substitution mutation mutants. The precise amino acid residues involved in the construction of the loopA, loopB and loopC mutants are highlighted in Fig. 4. All the constructs were generated from a plasmid template by a three-step genetic approach and confirmed by sequencing. After transformed into *S. mutans* UA159, the location and orientation of these constructs in the genome were further confirmed by a PCR strategy^30^. These mutants as well as control strains XT-C0 (ComD^+^) and Δ*comD* (ComD^−^) were then transformed with two *luxAB* reporter plasmids, pGF-PcipB and pGF-PnlmAB respectively, generating a set of *lux* reporter strains for luciferase reporter activity assays (Table S1). To detect the mutant loopA, loopB and loopC proteins in the membrane fractions by Western blotting, we also constructed a set of *S. mutans* strains that had a chromosomally deleted *comD* (Δ*comD*) but carried a shuttle vector pGF-Pldh-D-H that constitutively expressed a His-tagged loopA, loopB or loopC mutant protein. This shuttle vector was used to construct the strains, because it allowed not only rapid construction of the strains simply by replacing the wild copy *comD* between the P*ldh* promoter and 6His, but also constitutive expression of the mutant loopA, loopB and loopC proteins^31^. All the strains were then grown in THYE medium with addition of CSP to prepare the membrane proteins for Western blot analysis. The results showed that a strong reactive band (≈51-kDa) was detected in the membrane fractions of all the strains (Fig. 5), except the ComD^−^ mutant. The positive reactive bands in the membrane fractions of these strains were consistent with the size (50.5 kDa) of the wild type ComD protein^13^,^14^. The results clearly demonstrated that a short deletion or substitution mutation of these extracellular loops did not affect the translocation or insertion of these mutant proteins into the cytoplasmic membrane. Thus, all the mutants as well as control strains should be valid for studying the effects of a deletion or mutation of these extracellular loops on CSP perception and quorum sensing activation.

### Effects of a deletion or mutation of the extracellular loops on CSP perception

**Figure 6:**
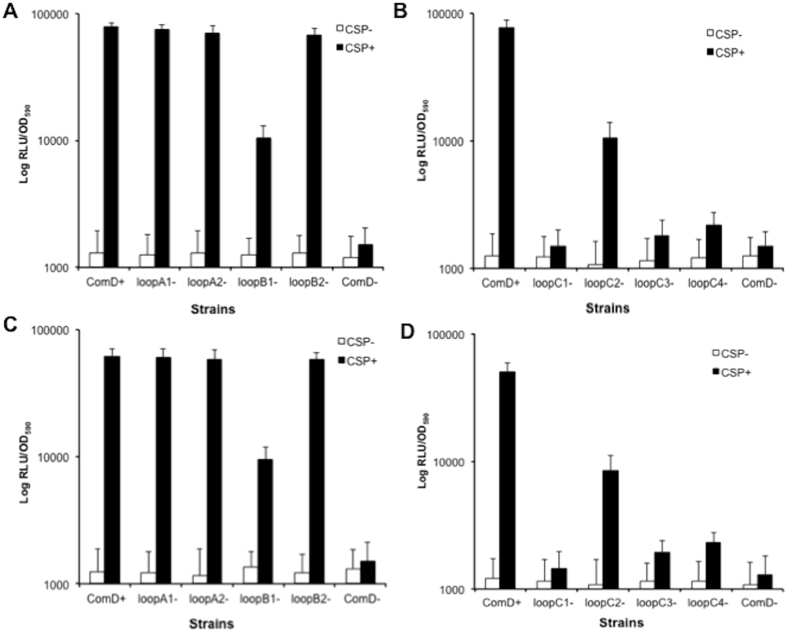
Effects of a deletion or mutation of loopA, loopB or loopC on CSP-dependent quorum-sensing activation. Two CSP-inducible promoters, P*cipB* and P*nlmAB*, were monitored for luciferase reporter activities. (**A**) Luciferase reporter activities (RLU/OD_590_) of P*cipB*::*luxAB* reporter strains, XT-Lx20 (ComD^+^), XT-Lx21 (loopA1^−^), XT-Lx22 (loopA2^−^), XT-Lx23 (loopB1^−^), XT-Lx24 (loopB2^−^), and XT-Lx29 (ComD^−^), were assayed with addition of CSP (CSP+) and without CSP (CSP−). (**B**) Luciferase reporter activities (RLU/OD_590_) of P*cipB*::*luxAB* reporter strains, XT-Lx20 (ComD^+^), XT-Lx25 (loopC1^−^), XT-Lx26 (loopC2^−^), XT-Lx27 (loopC3^−^), XT-Lx28 (loopC4^−^) and XT-Lx29 (ComD^−^), were assayed under the same conditions. (**C**) Luciferase reporter activities (RLU/OD_590_) of P*nlmAB*::*luxAB* reporter strains, XT-Lx30 (ComD^+^), XT-Lx31 (loopA1^−^), XT-Lx32 (loopA2^−^), XT-Lx33 (loopB1^−^), XT-Lx34 (loopB2^−^) and XT-Lx39 (ComD^−^), were assayed under the same conditions. (**D**) Luciferase reporter activities (RLU/OD_590_) of P*nlmAB*::*luxAB* reporter strains, XT-Lx30 (ComD^+^), XT-Lx35 (loopC1^−^), XT-Lx36 (loopC2^−^), XT-Lx37 (loopC3^−^), XT-Lx38 (loopC4^−^), and XT-Lx39 (ComD^−^), assayed under the same conditions.

Next, we determined whether a deletion or mutation of loopA, loopB or loopC affected CSP recognition and quorum sensing activation by examining specific luciferase reporter activities of the reporter strains in response to CSP. All the reporter strains were constructed in the mutant backgrounds and control strains (ComD^+^ and ComD^−^) by transforming a *luxAB* reporter plasmid, pGF-PcipB or pGF-PnlmAB into these strains. Therefore, each reporter strain carried a reporter plasmid containing a promoterless *luxAB* fused to the CSP-inducible promoter of two bacteriocin-encoding genes, *cipB* and *nlmAB*^18^. These two genes were chosen for constructing the reporter strains, because their promoters (P*cipB* and P*nlmAB*) contain the consensus ComE binding site and are directly controlled by the ComCDE quorum sensing system^18^,^32^. We then examined specific luciferase reporter activities of these strains in response to CSP. The results revealed that a deletion of loopA, either four residues SNVT (loopA1^−^) or eight residues SNVTLSKK (loopA2^−^), showed little effect on the responses of these two promoters to CSP, since the luciferase reporter activities in both loopA1^−^ and loopA2^−^ strains were similar to those in the ComD^+^ control strains (Fig. 6A,C). The results suggest that extracellular loopA appears to be not directly involved in CSP perception for quorum sensing activation. However, a deletion of four residues LDGT of loopB (loopB1^−^) resulted in a reduction in the luciferase reporter activities compared to the reporter activities in the ComD^+^ control strains. Interestingly, this was not the case when other four residues QGIV of loopB (loopB2^−^) were deleted, suggesting that residues LDGT but not QGIV of loopB largely participate in CSP recognition. Even more dramatically, a deletion of four residues NVIP of loopC (loopC1^−^) completely abolished the response of these two promoters to CSP, while a deletion of four residues TLKF of loopC (loopC2^−^) resulted in 50% of reduction in the *lux* reporter activities in response to CSP. This suggests that residues NVIP of loopC (loopC1^−^) may be essential for CSP recognition. The results strongly suggest that both extracellular loopC and loopB are required for CSP recognition and quorum sensing activation, while loopA appears to play little role in CSP detection.

To further confirm the results that the residues NVIP of loopC (loopC1^−^) may be essential for CSP perception, we constructed two more loopC mutants that had an alanine substitution mutation in loopC, designated as loopC3^−^ (AA/NV) and loopC4^−^ (AA/IP). These mutants were then transformed with the *lux* reporter plasmids, generating four more reporter strains. By assaying the specific reporter activities, we found that both substitution mutants failed to respond to CSP for induction of the luciferase reporter activities (Fig. 6B,D), confirming that the four residues NVIP of loopC are truly essential for CSP recognition. Importantly, these substitution mutations did not affect detection of the mutant proteins by Western blotting (Fig. 5C, lanes 3–4), suggesting no effect on the translocation or insertion of these mutant proteins into the cytoplasmic membrane. Thus the results confirm that at least four residues, NVIP, of loopC are essential for CSP recognition and quorum sensing activation in *S. mutans*.

### Natural point mutations in loopB or in both loopB and loopC on CSP perception

**Figure 7:**
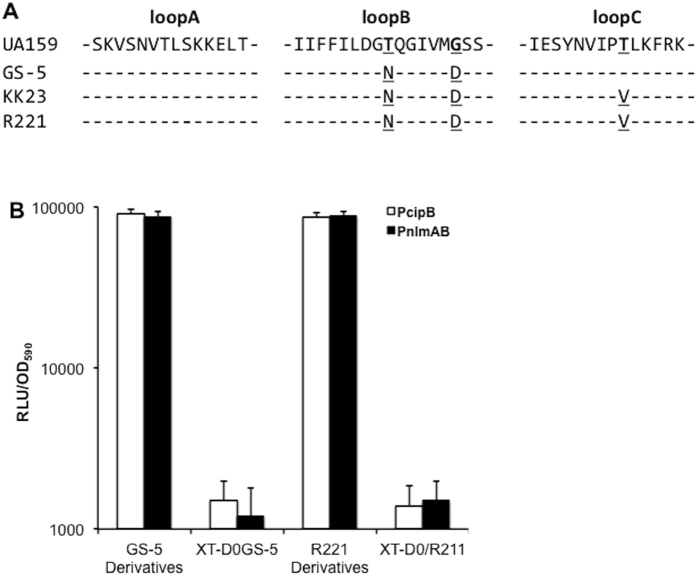
Effects of natural point mutations in loopB or in both loopB and loopC on CSP perception and quorum-sensing activation. (**A**) A sequence alignment of loopA, loopB and loopC among four *S. mutans* strains showing point mutations in loopB (T_110_ >N_110_ and G_116_ >D_116_) in strain GS-5 or in both loopB (T_110_ >N_110_ and G_116_ >D_116_) and loopC (T_178_ >V_178_) in strains KK23 and R221. (**B**) Luciferase report activities (RLU/OD_590_) of *S. mutans* GS-5-derived strains XT-Lx40 (white, P*cipB*) and XT-Lx41 (black, P*nlmAB*), XT-D0_GS5_ (ΔComD)-derived strains XT-Lx42 and XT-Lx43, R221-derived strains XT-Lx44 and XT-Lx45, and XT-D0_R211_ (ΔComD)-derived strains XT-Lx46 and XT-Lx47. The luciferase report activities (RLU/OD_590_) of P*cipB*::*luxAB* and P*nlmAB::luxAB* reporter strains were assayed in THYE medium with addition of CSP.

By sequence alignments of ComD proteins, we also identified several *S. mutans* strains that had one or two residue substitution mutations either within loopB, such as in strain GS-5, or within both loopB and loopC, such as strains KK23 and R221 (Fig. 7A). Compared with strain UA159, these strains have two amino acid residue substitutions of T_110_ (Threonine_110_) by N_110_ (Asparagine_110_) and G_116_ (Glycine_116_) by D_116_ (Aspartic acid_116_) within loopB. In addition to these two substitutions within loopB, strains KK23 and R221 also have one residue substitution of T_178_ (Threonine_178_) by V_178_ (Valine_178_) within loopC. We were curious to know whether these strains might be defective in perception and response to CSP for quorum sensing activation. Since both strains and their ComD deletion mutants were available in our lab, we directly used these strains to probe this question. We first transformed the *lux* reporter fusion plasmids, pGF-PcipB and pGF-PnlmAB, into these strains to generate two sets of new *lux* reporter strains (Table S1). We then assayed the luciferase reporter activities of these strains in response to CSP. Surprisingly, we found that all the strains showed the wild type levels of the luciferase reporter activities in response to CSP (Fig. 7B). There was no significant difference (*P* > 0.05) in the luciferase report activities between GS-5 or R221 and UA159 derived strains. In contrast, XT-D0_GS-5_ (ΔComD_GS-5_) and XT-D0_R211_ (ΔComD_R211_) derived reporter strains showed little induction in the luciferase report activities, suggesting that these mutants were unable to sense and respond to CSP, a pattern very similar to XT-D0_UA159_ (ComD^−^) derived reporter strains. The results suggest that the single residue mutations in loopB or in both loopB and loopC do not appear to affect CSP perception and quorum sensing activation in the *S. mutans* strains tested.

### Effects of a deletion or mutation of loopA, loopB or loopC on bacteriocin production

**Figure 8:**
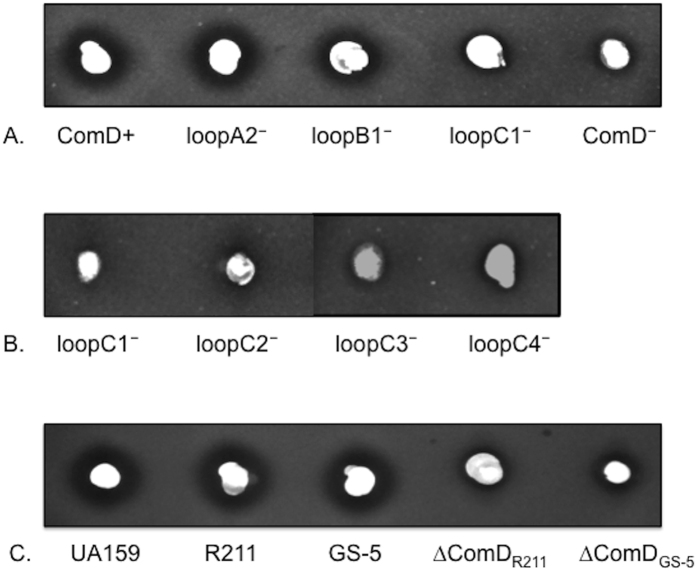
Effects of a deletion or mutation of loopA, loopB and loopC on bacteriocin production. A deferred antagonism assay was used to assess production of CSP-induced bacteriocins by *S. mutans* strains. The cell suspensions of each strain were stabled onto THYE agar plates and overlaid with an indicator strain *S. sanguinin* SK108 by mixing the cells (10^7^ CFU/ml) in low-melting agarose. The overlaid plates were incubated anaerobically at 37 °C for 20 hours before examining bacteriocin production. (**A**) ComD^+^ (XT-C0), loopA1^−^, loopB1^−^, loopC1^−^, ComD^−^ (ΔComD); (**B**) loopC1^−^, loopC2^−^, loopC3^−^, loopC4^−^; (**C**) UA159 (wt), R211 (wt), GS5 (wt), ΔComD_R211_ and ΔComD_GS-5_.

It is well known that CSP-mediated quorum sensing primarily regulates production of several bacteriocin-encoding genes, such as *cipB* (SMU.1904) and *nlmAB* (SMU.151/152) in *S. mutans*^18^. To determine the effects of a deletion or mutation of the extracellular loops on bacteriocin production, we examined CSP-inducible bacteriocin production of all the strains using a deferred antagonism assay. The results showed that except the loopA mutants that produced similar levels of bacteriocin to that by the ComD positive strain (ComD^+^), all the mutants produced reduced levels of bacteriocins compared to the ComD^+^ strain (Fig. 8). In particular, the loopC1, loopC3 and loopC4 mutants were significantly defective in producing bacterocins, which were consistent with the findings from the luciferase reporter activities. A minor inhibitory ring observed in the ComD deficient strains, including Δ*comD* mutant, might result from a bacteriocin that was not controlled by the ComCDE quorum sensing system^33^. The results confirm that a deletion or mutation of loopB and loopC resulted in moderate or severe deficiency in CSP-mediated bacterocin production. We also examined bacteriocin production of *S. mutans* strains GS-5 and R211 in response to CSP, since both strains have a residue substitution mutation either in loopB or in both loopB and loopC based on the genome sequences (Fig. 7A). The results showed that both strains produced nearly equal levels of bacteriocins to that by UA159. In contrast, both GS-5- and R211-derived ComD deletion mutants (ΔComD_GS-5_ and ΔComD_R211_) were defective in the production of bacteriocins (Fig. 8C). The results suggest that the single substitution mutations either in loopB or loopC appear to have little effect on CSP-dependent production of bacteriocins. The results are highly consistent with the previous reports showing that both strains GS-5 and R211 are potent bacteriocin producers^26^,^27^.

## Discussion

In Gram-positive bacteria, signal peptide pheromone-activated histidine protein kinases from the HPK_10_ subfamily control several important physiological processes, including competence development, bacteriocin production and virulence expression^3^,^10^,^11^. These signaling regulatory systems, including the *agr, com* and *pln* regulons, have been extensively studied with the respect to the events following phosphorylation of their cognate response regulators^10^,^11^,^23^,^24^. However, relatively little is known of interactions between signal pheromones and their cognate receptor proteins. No report has directly described ComD receptor kinase proteins, which are widely distributed among the members of the Genus *Streptococcus*^3^,^8^,^10^. In this study, we began to investigate the membrane topology of the *S. mutans* ComD protein, with our focus on the structural analysis of the extracellular loops of the ComD that acts as the CSP receptor. We demonstrate by the dual *phoA*-*lacZ* reporter system that the membrane-spanning domain of the ComD protein forms six transmembrane segments (TMSs) with three extracellular loops, loopA, loopB and loopC. The most important conclusion from the topology studies is that we confirm the extracellular locations of loopA, loopB and loopC. The results show very good agreement with the *in silico* predicted topology model of the ComD (Fig. 2), thereby, validating the membrane topology of the ComD receptor.

Upon the establishment of the membrane topology of ComD protein, we further explored the contribution of the extracellular loops, loopA, loopB and loopC, to CSP recognition and quorum-sensing activation, since the data obtained may have important implications for the ligand-receptor interaction and design of quorum sensing inhibitors. One of the major considerations in mutational analysis of the ComD protein was whether a partial deletion or mutation of these extracellular loops affects translocation or insertion of the mutant proteins into the membrane. It was important to track the mutant proteins in the membrane fractions, otherwise, it would be difficult to interpret the results regarding the effects of a deletion or mutation of the extracellular loops on CSP recognition and quorum sensing activation. To detect the mutant ComD proteins in the membranes by Western blotting, we constructed a new set of the *S. mutans* strains that constitutively expressed a His-tagged mutant loopA, loopB or loopC *in trans*. This ensured constitutive expression of the loopA, loopB and loopC mutant proteins in the chromosomally deleted *comD* mutant. Western blot analysis showed that all the mutant proteins (≈51 kDa) were detectable in the membrane fractions of these strains, suggesting that the mutant proteins could adequately translocate and insert into the membrane. Thus, the results from the luciferase reporter assays should be valid to evaluate the effects of a deletion or mutation of these loops on CSP recognition. Our work demonstrate that the extracellular loopC and loopB most likely form the receptor for CSP recognition, since a deletion or mutation of either loop resulted in partial or complete deficiencies in CSP-dependent quorum sensing activation. In contrast, loopA appears to play little role in signal detection, because a deletion of up to eight residues of this loop caused little effect on quorum sensing activation. In addition, we have confirmed that at least four residues, NVIP, of loopC are essential for CSP recognition, since a deletion or mutation of these residues abolishes CSP recognition and quorum sensing activation.

Another interesting finding from this study is that by sequence alignments of the *S. mutans* ComD proteins we have identified several *S. mutans* strains that have one or two point substitution mutations either within loopB, such as in strain GS-5, or within both loopB and loopC, such as in strain R221. However, our experiments confirm that both of these strains can detect and respond to CSP as effectively as *S. mutans* UA159. The results reveal that these single substitution mutations, even at such important locations of the receptor, do not appear to affect CSP recognition and quorum sensing activation. Neither do these mutations significantly affect CSP-induced bacteriocin production, suggesting that the ComD receptor in *S. mutans* displays relatively low specificity to sense the signal for quorum sensing activation within the species. Such less constraint specificity in CSP-ComD interaction in *S. mutans* strains clearly differs from those in *S. aureus* and *S. pneumoniae*. It has been well recognized that quorum-sensing signaling molecules produced by many bacteria often induce species-specific or even strain-specific activities at nano-molar concentrations. This feature has been used to explore structure-activity relationships between a signal pheromone and its cognate receptor^12^,^16^,^21^,^34^. For example, the AIP-AgrC quorum sensing system in *S. aureus* is one of the best-studied model systems that show highly strain-specific activities to induce quorum-sensing response^11^,^34^. The sequence variations of the AIPs from different *S. aureus* strains have led to at least four specificity groups in *S. aureus*^23^,^35^. All strains within one group produce the same AIP, which only activates quorum sensing and the virulence within its own specificity group but not in other groups. In *S. pneumoniae*, extensive screening of pneumococcal isolates reveals two major CSP variants that are highly specific to interact with their respective receptors, ComD1 and ComD2^36^,^37^. These studies have led to the proposal that *S. pneumoniae* strains can be divided into different pherotypes based on their quorum sensing pheromone specificity^10^,^36^. Similar studies have been carried out to identify quorum-sensing signaling peptide variants from *S. mutans* strains and clinical isolates. Seven *comC* alleles encoding three distinct mature CSPs are identified among 36 geographically diverse *S. mutans* strains^15^. In contrast to *S. pneumoniae*, however, all three CSP variants function equally well to induce quorum sensing, bacteriocin production and genetic competence^15^,^17^,^21^. There is no evidence showing quorum sensing signal pheromone pherotype in *S. mutans*. In fact, structural and functional divergences in ComCDE quorum sensing systems between *S. mutans* and *S. pneumoniae* have been recognized for some years^38^. Phylogenetic analysis of various streptococcal genomes reveals that ComCDE system in *S. mutans* is more closely related to BlpCRH quorum sensing system that directly controls bacteriocin production in *S. pneumoniae*^38^,^39^. Our sequence alignments also show that ComD proteins of *S. mutans* only shares 22% of identity and 44% of similarity with those of *S. pneumoniae*. This suggests that ComD proteins of *S. mutans* may not necessarily share the same membrane topology and signal recognition domains with ComD proteins in *S. pneumoniae*. Despite no experiments performed to test this difference, *in silico* analysis of ComD proteins of *S. pneumoniae* strains appears to support this suggestion^10^. The ComCDE system in *S. mutans* is suggested to combine the action of two orthologous systems, the ComCDE and BlpCRH in *S. pneumoniae,* which are well known to be involved in competence development and bacteriocin production, respectively^38^. What is the ecological implication of such less constraint specificity of the ComD-CSP interaction in *S. mutans* is unclear. However, we speculate that the less constraint specificity may provide *S. mutans* with more flexibility or even an advantage to sense the signal molecule for intra-species communication in densely packaged dental biofilms, where *S. mutans* needs to compete with closely related colonizers, such as various species of streptococci, by population-wide production of bacteriocins. This speculation regarding the ComCDE controlled activities of *S. mutans* in dental biofilms is currently under our investigation.

In summary, this study demonstrates that the membrane domain of the *S. mutans* ComD protein forms six transmembrane segments and three extracellular loops, loopA, loopB and loopC. Structural analysis of these extracellular loops reveals that both loopC and loopB are required for CSP recognition and quorum sensing activation, while loopA plays little role in CSP detection. However, single sequence variations in loopB and loopC exist, such as in *S. mutans* strains GS-5 and R211, but these strains do not show any detectable defects in CSP recognition. Thus, unlike other pheromone receptors in the HPK_10_ subfamily, the ComD receptor in *S. mutans* shows more flexibility to sense and transduce the signal for quorum sensing activation, which may provide *S. mutans* with an advantage for intra-species communication in its natural ecosystem, dental biofilms.

## Methods

### Bacterial strains, media and growth conditions

Bacterial strains and plasmids used in this study are listed in Tables S1 and S2, respectively. *S. mutans* wild-type strains UA159, GS-5 and R211 were grown on Todd-Hewitt medium supplemented with 0.3% yeast extract (THYE), whereas all the mutants, the *lux* reporter strains and other strains derived from *S. mutans* UA159, GS-5 and R211 were maintained on THYE medium supplemented with an appropriate antibiotic(s). *Escherichia coli* hosts and their derivatives generated by molecular cloning were grown in Luria-Bertani (LB) medium supplemented with an appropriate antibiotic(s).

### *In silico* prediction of ComD topology

We combined several topology prediction methods, including SOSUI (http://bp.nuap.nagoya-u.ac.jp/sosui/), SMART (http://smart.embl-heidelberg.de/smart/), TMHMM (http://www.cbs.dtu.dk/services/TMHMM-2.0/) and PSIPRED (http://bioinf.cs.ucl.ac.uk/psipred/), to obtain a hypothetical topology model of ComD protein from *S. mutans* UA159. All the methods were used in single protein mode and user-adjustable parameters were left at their default values. Such *in silico* analyses of the ComD protein had led us to generate a hypothetical topology model (Fig. 2), which facilitated the design and genetic construction of dual reporter fusion strains and mutants. In addition, the sequence alignments of ComD proteins from many strains of *S. mutans* and *S. pneumoniae* were performed with MacVector 9.0 ClusterW. All the protein sequences of ComD proteins from these strains were obtained from the NCBI protein database (http://www.ncbi.nlm.nih.gov/protein/).

### Construction of *comD-phoA-lacZ* fusion reporters

To study the membrane topology of ComD protein, we used a dual *phoA*-*lacZ* reporter system, pKTop, which consists of an *E. coli* alkaline phosphatase fragment PhoA_22-472_ fused in frame after the α-fragment of β-galactosidase LacZ_4-60_^40^, to construct a series of *comD*-*phoA*-*lacZ* fusion reporters in frame after selected *comD* codons, including L38 (pKTop-ComD_1-L38_), A70 (pKTop-ComD_1–A70_), T110 (pKTop-ComD_1–T110_), S150 (pKTop-ComD_1–S150_), P187 (pKTop-comD_1–P187_) and A224 (pKTop-ComD_1–A225_). The corresponding *comD* fragments were amplified from the *S. mutans* UA159 genomic DNA by PCR using appropriate primer pairs (Table S3) and subcloned into the BamHI and KpnI restriction sites of pKTop^28^. Positive transformants grown on LB plates supplemented with kanamycin (50 μg/ml) were selected for genetic confirmation by PCR and restriction analysis. For the membrane protein topology assays, *E. coli* DH5α were transformed with the resulted fusion plasmids to generate six pKTop derivatives expressing tripartite ComD*-*PhoA/LacZ. The *phoA* and *lacZ* genes missing their leader sequence were used as negative control for the report activity assays. The resulting reporter strains were then grown on LB plates containing dual indicators of a blue chromogenic substrate, 5-bromo-4-chloro-3-indoxyl phosphate disodium salt or X-Pho (Sigma) at a concentration of 80 μg/ml, and a red chromogenic substrate, 6-chloro-3-indoxyl-β-D-galactopyranoside or Salmon-Gal (Gold Biotech) at a concentration 120 μg/ml and IPTG (1 mM) with kanamycin (50 μg/ml). The periplasmic or cytosolic location was determined based on the locations of these fusion reporters. A periplasmic or extracellular location of a reporter fusion point should lead to the higher alkaline phosphatase activity (blue color), whereas a cytosolic location of a reporter fusion point should result in the higher β-galactosidase activity (pink color)^28^,^40^.

### Construction of extracellular loopA, loopB and loopC mutants

To evaluate the contribution of the ComD extracellular loops to CSP recognition, we constructed eight *comD* mutants by a three-step genetic approach (Fig. S1). Each mutant constructed had an in-frame deletion or substitution mutation in loopA, loopB or loopC. In the first step, a 2064-bp DNA fragment containing the *comD*-coding sequence and its flanking regions was amplified by PCR against the genomic DNA of *S. mutans* UA159, and subcloned into pBlueScript SKII (Stratagene). The resulting plasmid was then used as a template to generate an amplicon (the entire plasmid) with two restriction sites of *Asc*I and *Fse*I by circular PCR from the location downstream of *comD* but within *comC*. The amplicon was digested and ligated to the same restriction sites of an erythromycin resistance cassette. The ligation product was cloned into an *E. coli* host, generating a new plasmid, pGF-C0 that contained a wild copy of *comD* (ComD^+^) but with *comC* (ComC^−^) inactivated by the insertion of the *erm* cassette. In the second step, this new plasmid was used as a template to generate six deletion constructs of loopA, loopB and loopC by an inverse PCR strategy and two alanine substitution mutant constructs corresponding to the target codons of loopC by a QuickChange II site-directed mutagenesis kit (Agilent Tech. Inc.). The resulting products were digested with *BamH*1, self-ligated and cloned into *E. coli*, generating new plasmids that harbored a mutant construct of loopA, loopB and loopC, respectively. All plasmids with a mutant construct were genetically confirmed by sequencing. In the last step, these plasmids were linearized and transformed into *S. mutant* UA159. Following double-crossover recombination, each of these constructs was integrated into the *S. mutans* genome, generating a number of ComD mutants (Table S1). Positive transformants were selected from THYE plates plus erythromycin (10 μg/ml) and confirmed by a PCR strategy using a combination of four primers specific to *comD* and the *erm* cassette^30^. A *S. mutant* strain with a full-length ComD but with *comC* inactivated by an insertion of the *erm* cassette (ComD^+^, ComC^−^, Erm^r^) was included as a positive control, while *comD* deletion mutant (ΔcomD) was used as a negative control. Since the *comC* was inactivated by the insertion of the *erm* cassette, all the strains constructed were unable to produce endogenous CSP.

### Construction of *S. mutans* strains expressing a His-tagged, ComD mutant protein

To determine whether a deletion or point mutation of loopA, loopB or loopC affected translocation or insertion of the mutant proteins into the cytoplasmic membrane, we also constructed a set of *S. mutans* strains that carried a shuttle vector expressing a His-tagged ComD mutant protein under control of a constitutively expressed promoter of *ldh,* the gene encoding lactate dehydrogenase in *S. mutans*^13^,^31^. This allowed subsequent detection of His-tagged mutant proteins by Western blotting. To achieve this goal, we simply amplified each of the ComD mutant constructs (except stop codon) from constructed plasmids by primers ComD-BamHI-F and ComD-NotI-B. The PCR products were digested, purified and cloned into a vector pGF-Pldh-D-H by replacing the *comD* (Fig. S2). Each plasmid was constructed in such a way that the coding sequence was fused to the *ldh* promoter (P*ldh*) with its start codon and to 6His-tag with its end, generating a number of plasmids (Table S2). A plasmid pGF-Pldh-H (without *comD*) was also generated as a negative control by removing the *comD* from pGF-Pldh-D-H. The newly constructed plasmids were then confirmed by PCR and sequencing. The confirmed plasmids were transformed into the Δ*comD* mutant, generating a new set of *S. mutans* strains that constitutively expressed a His-tagged mutant ComD protein that could be detected in the membrane fractions by Western blotting.

### Cell fractionation and protein detection by Western blotting

The *S. mutans* strains that expressed a His-tagged mutant *comD* were grown in THYE medium. When reaching to the early-log phase (OD_600_ ≈ 0.3), all the cultures were added with 1 μM of CSP and incubated until the mid-log phase (OD_600_ ≈ 0.6). Aliquots of samples were taken to prepare the crude membrane proteins using a modified method as described previously^41^,^42^. Briefly, the cell pellets were re-suspended in 2 ml of TE buffer (50 mM Tris-HCl, 50 mM EDTA, pH 7.6, 200 μg/ml of lysozyme and 30 μg/ml of mutalysin) and incubated at 37 °C for 2 hrs. The cell lysates were prepared by sonication on ice at 30 s pulses for 3 times after added with an aliquot of a protease inhibitor cocktail (Sigma-Aldrich). The cell lysates were centrifuged at 30, 000× *g* at 4 °C for 1 h. The membrane fractions were resuspented in 100 μl of 50 mM ammonium bicarbonate (pH 11) and the proteins were extracted with 100 μl of trifluroethanol/chloroform (2:1 v/v)^41^,^42^. The samples were resuspended in 60 μl of solubilization buffer (7M urea, 2M thiourea, 2% Triton X-100, 0.5% ASB-14, 50 mM dithiothreitol, 0.2% Bio-Lytes). The proteins were resolved on SDS-PAGE gels and transferred to polyvinylidene difluoride (PVDF) membranes for Western blot analysis using an anti-His-tag antibody (1:3,000 dilution) by the method as described previously^43^. The membranes were washed and detected with an AP detection reagent (Novagen). The proteins on the membrane were analyzed using FluorChem SP image system (Alpha Innotech, Calif.).

### Construction of *luxAB* transcriptional reporter strains and luciferase reporter assay

To determine the effects of a deletion or mutation of the extracellular loops on CSP perception and quorum sensing activation, we construct a number of transcriptional *luxAB* reporter strains in the loopA, loopB and loopC mutant backgrounds (Fig. S3). Each strain harbored a shuttle vector that carried a promoterless *luxAB* fused to the promoter of two CSP-inducible, bacteriocin-encoding gene, *cipB* and *nlmAB*^13^,^18^. The DNA fragments containing the promoter regions of these genes were generated by PCR, purified and cloned into a shuttle vector pGF-kan^43^, generating two *luxAB* reporter fusion plasmids (Table S2). The plasmids were genetically confirmed by PCR and sequencing. The confirmed plasmids, designated pGF-PcipB and pGF-PnlmAB, were transformed into the *S. mutans* mutants and control strains (ComD^+^ and ComD^−^), generating two groups of *luxAB* reporter strains that were then used to assay specific luciferase reporter activities of the promoters, P*cipB* and P*nlmAB*, in response to CSP.

To determine the effects of a deletion or mutation of loopA, loopB or loopC on CSP perception and quorum sensing activation, luciferase reporter activity assays were carried out to examine CSP-dependent quorum sensing activation. All the mutants that carried either pGF-PcipB or pGF-PnlmAB were grown to assay their specific luciferase report activities in response to CSP (SGSLSTFFRLFNRSFTQA), which was synthesized with 90% purity commercially (BioBasic Inc., Ontario). CSP was freshly dissolved in sterile distilled water at 1.0 mM (stock), which was further diluted to make working concentrations as required. *S. mutans* wild type background strains carrying the reporter plasmid were used as positive controls (ComD^+^), whereas the ΔcomD background strains carrying the plasmids were used as negative controls (ComD^−^). All the strains were grown in THYE broth and the cultures were added with an aliquot of CSP (1 μM) at O.D_590_ ≈ 0.3. Bacterial growth and luciferase reporter activities (O.D_590_) of the strains were assayed with a 96-well microplate reader (Synergy HT, Biotek, USA). One percent nonanal (Sigma-Aldrich) was added into the cultures, since LuxAB-catalyzed luciferase activity requires nonanal as a substrate^31^. Briefly, aliquots (200 μl) of cell suspension were transferred to wells of a pre-warmed (37 °C) microtiter plate. A 50 μl of the solution containing 1% nonanal in mineral oil in a volatile form were placed in the spaces of wells and the plate was covered with a lip. The microtiter plates were incubated and read in the prewarmed (37 °C) microplate reader at O.D_590_ at a time intervals of 15 min for 4 hours. The results were expressed in relative luminescent units (RLU) divided by cell density of the cultures. The data plotted represented the maximal RLU readings of each strain.

### Deferred antagonism assay

To determine the effects of a deletion or mutation of loopA, loopB or loopC on CSP-induced bacteriocin production, all the *S. mutans* strains were grown for overnight. Aliquots of the cell suspension were stabbed onto THYE agar, incubated anaerobically at 37 °C for six hours and added on the surfaces of each inoculum with 5 μl of CSP (1 μM). The agar plates were then overlaid with a freshly grown indicator strain *S. sanguinis* SK108 by mixing the cell suspension (at 10^7^ CFU/ml) with low-melting agarose (Bishop Canada Inc.). The overlaid plates were incubated anaerobically at 37 °C for additional 20 hours before inspection of bacteriocin production. *S. mutans* strains showing inhibitory zones around the stabs indicated positive production of bacteriocins^21^.

## Additional Information

**How to cite this article**: Dong, G. *et al*. Membrane Topology and Structural Insights into the Peptide Pheromone Receptor ComD, A Quorum-Sensing Histidine Protein Kinase of *Streptococcus mutans. Sci. Rep.* **6**, 26502; doi: 10.1038/srep26502 (2016).

## Supplementary Material

Supplementary Information

## Figures and Tables

**Figure 1:**
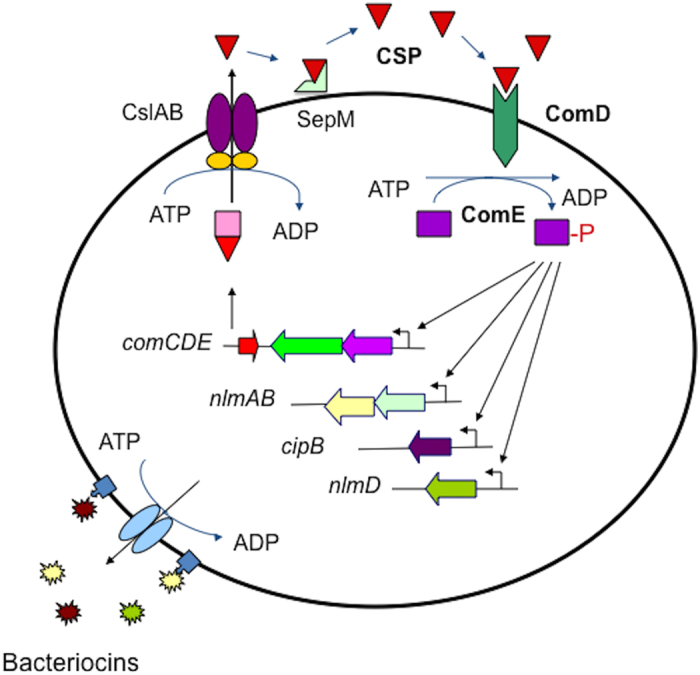
A schematic diagram describes the ComCDE quorum sensing system and its regulated genes in *S. mutans*. The *comC* encodes a signal peptide precursor, which is cleaved and exported to release a 21-residue peptide through a peptide-specific ABC transporter encoded by *cslAB*. The 21-aa peptide is further modified by an extracellular protease SepM to remove the *C*-terminal 3 residues and generate an 18-residue functional peptide or competence-stimulating peptide (CSP). The *comDE* encode a two-component transduction system that specifically senses and responds to CSP. When it reaches a critical concentration, CSP interacts with the ComD receptor protein of the neighboring cells and activates its cognate response regulator, ComE, through autophospharylation. The phospharylated ComE in turn activates downstream genes, triggering the signaling cascade for bacteriocin production and other cell density-dependent activities.
