# Supplementary material for: Membrane Topology and Structural Insights into the Peptide Pheromone Receptor ComD, A Quorum-Sensing Histidine Protein Kinase of Streptococcus mutans
Source: Sci Rep. 2016 May 20;6:26502. doi: 10.1038/srep26502 (PMC4873836; doi:10.1038/srep26502)
Supplement: Supplementary Information [file srep26502-s1.doc]

**Membrane Topology and Structural Insights into the Peptide Pheromone Receptor ComD, A Quorum-Sensing Histidine Protein Kinase of *Streptococcus mutans***

Gaofeng Dong ^a,§^, Xiao-Lin Tian ^a,§^, Kayla Cyr ^a^, Tianlei Liu ^a^, William Lin ^b^, Geoffrey Tziolas ^b^, and Yung-Hua Li ^a,b^,*

Department of Applied Oral Sciences ^a^

Department of Microbiology and Immunology ^b^

Dalhousie University

Halifax, NS, Canada

**Supplementary Information**

***: Corresponding Author**

Yung-Hua Li

5981 University Ave. Rm5215

Halifax, Nova Scotia

Canada, B3H 1W2

Tel: 1-902-494-3063

Fax: 1-902-494-6621

E-mail: [yung-hua.li@dal.ca](mailto:yung-hua.li@dal.ca)

**§:** G.D and X.L.T contributed equally to this work.

## Table S1. Bacterial Strains used in This Study

| Strains | Relevant Characteristics | Source/ref. |
| --- | --- | --- |
| *E. coli* |  |  |
| DH5α | Cloning host | Invitrogen |
| GF-pKTop | DH5α carrying pKTop dual reporter vector, Kan^r^ | This study |
| GF-L38 | DH5α carrying pGF-DL38, Kan^r^ | This study |
| GF-A70 | DH5α carrying pGF-DA70, Kan^r^ | This study |
| GF-T110 | DH5α carrying pGF-DT110, Kan^r^ | This study |
| GF-S150 | DH5α carrying pGF-DS150, Kan^r^ | This study |
| GF-P187 | DH5α carrying pGF-DP187, Kan^r^ | This study |
| GF-A224 | DH5α carrying pGF-DA224, Kan^r^ | This study |
|  |  |  |
| *S. mutans comD* mutants | |  |
| UA159 (wt) | Wild type, the genome sequence reference strain | 13 |
| XT-C0 | UA159 but *comC79::erm*, *comC*^−^, *comD*^+^, Em^r^ | This study |
| loopA1^−^ | XT-C0 but loopA_∆S29-T32_, *comC::erm*, *comC*^−^, Em^r^ | This study |
| loopA2^−^ | XT-C0 but loopA_∆29-K36_, *comC::erm*, *comC*^−^, Em^r^ | This study |
| loopB1^−^ | XT-C0 but loopB_∆L107-T110_*, comC::erm*, *comC*^−^, Em^r^ | This study |
| loopB2^−^ | XT-C0 but loopB_∆Q111-V114_*, comC::erm*, *comC*^−^, Em^r^ | This study |
| loopC1^−^ | XT-C0 but loopC_∆N184-P187_*, comC::erm*, *comC*^−^, Em^r^ | This study |
| loopC2^−^ | XT-C0 but loopC _∆L188-F191_*, comC::erm*, *comC*^−^, Em^r^ | This study |
| loopC3^−^ | XT-C0 but loopC_NV/AA(184-185)_*, comC::erm*, *comC*^−^, Em^r^ | This study |
| loopC4^−^ | XT-C0 but loopC_IP/AA(186-187)_*, comC::erm*, *comC*^−^, Em^r^ | This study |
| ΔComD | UA159 but Δ*comD, comD*^−^, Em^r^ | This study |
|  |  |  |
| *S. mutans comD* mutants carrying a shuttle vector pGF-D-H | |  |
| XT-Pldh-D-H | ΔComD carrying pGF-*comD*::His, Spec^r^ | This study |
| XT-A1H | ΔComD carrying pGF-Pldh::loopA1_∆S29-T32_::His, Spec^r^ | This study |
| XT-A2H | ΔComD carrying pGF-Pldh::loopA2_∆S29-K36_::His, Spec^r^ | This study |
| XT-B1H | ΔComD carrying pGF-Pldh::loopB1_∆L107-T110_::His, Spec^r^ | This study |
| XT-B2H | ΔComD carrying pGF-Pldh::loopB2_∆Q111-V114_::His, Spec^r^ | This study |
| XT-C1H | ΔComD carrying pGF-Pldh::loopC1_∆N184-P187_::His, Spec^r^ | This study |
| XT-C2H | ΔComD carrying pGF-Pldh::loopC2_∆L188-F191_::His, Spec^r^ | This study |
| XT-C3H | ΔComD carrying pGF-Pldh::loopC3 _NV/AA(184-185)_::His, Spec^r^ | This study |
| XT-C4H | ΔComD carrying pGF-Pldh::loopC4 _IP/AA(186-187)_::His, Spec^r^ | This study |
| XT-Pldh-H | ΔComD carrying pGF-Pldh::His, *comD*^−^, Spec^r^ | This study |
|  |  |  |
| *S. mutans comD* mutants carrying a *lux* reporter plasmid | |  |
| XT-Lx20 | XT-C0 carrying pGF-PcipB, Em^r^, Kan^r^ | This study |
| XT-Lx21 | XT-loopA1 carrying pGF-PcipB, Em^r^ Kan^r^ | This study |
| XT-Lx22 | XT-loopA2 carrying pGF-PcipB, Em^r^ Kan^r^ | This study |
| XT-Lx23 | XT-loopB1 carrying pGF-PcipB, Em^r^ Kan^r^ | This study |
| XT-Lx24 | XT-loopB2 carrying pGF-PcipB, Em^r^ Kan^r^ | This study |
| XT-Lx25 | XT-loopC1 carrying pGF-PcipB, Em^r^ Kan^r^ | This study |
| XT-Lx26 | XT-loopC2 carrying pGF-PcipB, Em^r^ Kan^r^ | This study |
| XT-Lx27 | XT-loopC3 carrying pGF-PcipB, Em^r^ Kan^r^ | This study |
| XT-Lx28 | XT-loopC4 carrying pGF-PcipB, Em^r^ Kan^r^ | This study |
| XT-Lx29 | ΔcomD (ComD^−^) carrying pGF-PcipB, Em^r^ Kan^r^ | This study |
| XT-Lx30 | XT-C0 carrying pGF-PnlmAB, Em^r^ Kan^r^ | This study |
| XT-Lx31 | XT-loopA1 carrying pGF-PnlmAB, Em^r^ Kan^r^ | This study |
| XT-Lx32 | XT-loopA2 carrying pGF-PnlmAB, Em^r^ Kan^r^ | This study |
| XT-Lx33 | XT-loopB1 carrying pGF-PnlmAB, Em^r^ Kan^r^ | This study |
| XT-Lx34 | XT-loopB2 carrying pGF-PnlmAB, Em^r^ Kan^r^ | This study |
| XT-Lx35 | XT-loopC1 carrying pGF-PnlmAB, Em^r^ Kan^r^ | This study |
| XT-Lx36 | XT-loopC2 carrying pGF-PnlmAB, Em^r^ Kan^r^ | This study |
| XT-Lx37 | XT-loopC3 carrying pGF-PnlmAB, Em^r^ Kan^r^ | This study |
| XT-Lx38 | XT-loopC4 carrying pGF-PnlmAB, Em^r^ Kan^r^ | This study |
| XT-Lx39 | ΔcomD (ComD^−^) carrying pGF-PnlmAB, Em^r^ Kan^r^ | This study |
|  |  |  |
| Other *S. mutans* strains carrying a *lux* reporter plasmid | |  |
| GS-5 | Wild type strain, serotype C | 26 |
| XT-Lx40 | GS5 carrying pGF-PcipB, Kan^r^ | This study |
| XT-Lx41 | GS5 carrying pGF-nlmAB, Kan^r^ | This study |
| XT-D0_GS5_ | GS5 but Δ*comD::erm*, Erm^r^ | This study |
| XT-Lx42 | XT-D0_GS5_ carrying pGF-PcipB, Em^r^ Kan^r^ | This study |
| XT-Lx43 | XT-D0_GS5_ carrying pGF-PnlmAB, Em^r^ Kan^r^ | This study |
| R221 | Clinical strain, serotype C | 27 |
| XT-Lx44 | R211 carrying pGF-PcipB, Kan^r^ | This study |
| XT-Lx45 | R211 carrying pGF-PnlmAB, Kan^r^ | This study |
| XT-D0_R211_ | R211 but Δ*comD::erm*, Erm^r^ | This study |
| XT-Lx46 | XT-D0_R211_ carrying pGF-PcipB, Em^r^ Kan^r^ | This study |
| XT-Lx47 | XT-D0_R211_ carrying pGF-PnlmAB, Em^r^ Kan^r^ | This study |
|  |  |  |

## Table S2 Plasmids used in This Study

| Strains | Relevant Characteristics | Source/ref. |
| --- | --- | --- |
| **Plasmid** |  |  |
| pKTop | A dual reporter vector, PhoA_22-472_/LacZ_4-60_, p15 ori, Kan^r^ | 28 |
| pGF-L38 | pKTop derivative expressing ComD_1–L38_/PhoA/LacZ, Kan^r^ | This study |
| pGF-A70 | pKTop derivative expressing ComD_1–A70_/PhoA/LacZ, Kan^r^ | This study |
| pGF-T110 | pKTop derivative expressing ComD_1–T110_/PhoA/LacZ, Kan^r^ | This study |
| pGF-S150 | pKTop derivative expressing ComD_1–S150_/PhoA/LacZ, Kan^r^ | This study |
| pGF-P187 | pKTop derivative expressing ComD_1–P187_/PhoA/LacZ, Kan^r^ | This study |
| pGF-A224 | pKTop derivative expressing ComD_1–A224_/PhoA/LacZ, Kan^r^ | This study |
|  |  |  |
| pBlueScriptSKII | Cloning vector, Amp^r^ | Stratagene |
| pGF-comD | pBlueScript II SK::*comD-*2064; Amp^r^ | This study |
| pGF-C0 | pGF-D(wt) having *comC79::erm*, ComC^−^, Amp^r^, Em^r^ | This study |
| pGF-A1 | pGF-D/C0 having loopA_∆S29-T32_; ComC^−^, Amp^r^, Em^r^ | This study |
| pGF-A2 | pGF-D/C0 having loopA_∆S29-K36_; ComC^−^, Amp^r^, Em^r^ | This study |
| pGF-B1 | pGF-D/C0 having loopB_∆L107-T110_*, comC::erm*, *comC*^−^, Em^r^ | This study |
| pGF-B2 | pGF-D/C0 having loopB_∆Q111-V114_*, comC::erm*, *comC*^−^, Em^r^ | This study |
| pGF-C1 | pGF-D/C0 having loopC_∆N184-P187_; ComC^−^, Amp^r^, Em^r^ | This study |
| pGF-C2 | pGF-D/C0 having loopC_∆L188-F191_*, comC::erm*, *comC*^−^, Em^r^ | This study |
| pGF-C3 | pGF-D/C0 having loopC_NV/AA(184-185)_*, comC::erm*, *comC*^−^, Em^r^ | This study |
| pGF-C4 | pGF-D/C0 having loopC_IP/AA(186-187)_*, comC::erm*, *comC*^−^, Em^r^ | This study |
|  |  |  |
| pWAR303 | A shuttle vector containing promoterless *luxAB*, Em^r^ | 43 |
| pGF-kan | pWAR303 but the *erm* cassette replaced by *kan*, Kan^r^ | This study |
| pGF-PcipB | pGF-kan having a fusion of P*cipB::luxAB*; Kan^r^ | This study |
| pGF-PnlmAB | pGF-kan having a fusion of P*nlmAB::luxAB*; Kan^r^ | This study |
|  |  |  |
| pGF-Pldh-D-H | pXTH-Pldh::*comD*::6His, Spec^r^ | This study |
| pGF-Pldh-H | pDL277::P*ldh*::6His, *comD*^−^, Spec^r^ | This study |
| pXT-Pldh | pDL277::promoter of *ldh* (Pldh), Spec^r^ | 31 |
| pET20-D-H | pET20b::*comD* with C-terminal 6His-tag; Spec^r^ | This study |
| pET20b(+) | Expression vector; Amp^r^; | Novagen |
| pGF-A1-H | pGF-Pldh::XDloopA1_∆S29-T32_::His, Spec^r^ | This study |
| pGF-A2-H | pGF-Pldh::XDloopB1_∆S29-K36_::His, Spec^r^ | This study |
| pGF-B1-H | pGF-Pldh::XDloopB2_∆L107-T110_::His, Spec^r^ | This study |
| pGF-B2-H | pGF-Pldh::XDloopB3_∆Q111-V114_::His, Spec^r^ | This study |
| pGF-C1-H | pGF-Pldh::XDloopC1_∆N184-P187_::His, Spec^r^ | This study |
| pGF-C2-H | pGF-Pldh::XDloopC2_∆L188-F191_::His, Spec^r^ | This study |
| pGF-C3-H | pGF-Pldh::XDloopC3_NV/AA(184-185)_::His, Spec^r^ | This study |
| pGF-C4-H | pGF-Pldh::XDloopC4 _IP/AA(186-187)_::His, Spec^r^ | This study |
|  |  |  |

**Table S3 Primers used in This Study**

| **Primers*** | **Nucleotide Sequence (5’ -> 3’)** | **Purpose** |
| --- | --- | --- |
|  |  |  |
| ComD-F | GCG**GGATCC**CATGAATGAAGCCTTAATGATAC |  |
| ComD-L38-B | CGC**GGTACC**TCTAATTCCTTTTTCGATAAAGTG | ComD_1-38_ |
|  |  |  |
| ComD-A70-B | CGC**GGTACC**TCAGCTATAAAATAAAGAGGCTCTG | ComD_1-70_ |
|  |  |  |
| ComD-T110-B | CGC**GGTACC**TCAGTTCCATCCAAGATAAAGAA | ComD_1-110_ |
|  |  |  |
| ComD-S150-B | CGC**GGTACC**TCACTATCTTTAAGTCGACCAATATC | ComD_1-150_ |
|  |  |  |
| ComD-P187-B | CGC**GGTACC**TCCGGTATCACATTATAACTCTCTAT | ComD_1-187_ |
|  |  |  |
| ComD-A224-B | CGC**GGTACC**TCTGCCATTATCTCATTTTGAA | ComD_1-224_ |
|  |  |  |
| ComD-up-F  ComD-dw-B | CG**CTCGAG**GATTGAATTCTACGGCAGTATG GC**TCTAGA**ATTTTTAGTTTTTTGTCTGGCTG | comD and flanking |
|  |  |  |
| ComDc-up-F  ComDc-dw-B | TA**GGCGCGCC**GGAAGCCTATCAACATTTTTC  GCTGGCCGGCCGCTTCCGCCAATGATAATCT | inverse PCR-comDc |
|  |  |  |
| ComD-inv-B  ComD-inv-F | TA**GGCGCGCC**CTGTCGTGGATGAGAAGATAA  GCT**GGCCGGCC**CACACTTGATTATTTAACCCTGT | inverse PCR-comD |
|  |  |  |
| Erm-F  Erm-B | TA**GGCGCGCC**CCGGGCCCAAAATTTGTTTGAT  GCT**GGCCGGCC**AGTCGGCAGCGACTCATAGAA | erm  cassette |
|  |  |  |
| Kan-F  Kan-B | CG**GGATCC**GACGAAGAGGATGAAGAGGAT  AA**GCGGCCGC**AAGTAGTTTCCGATATGGACG | kan  cassette |
|  |  |  |
| ComD-BamHI-F  ComD-NotI-B | CG**GGATCCG**ATGAATGAAGCCTTAATGATA AA**GCGGCCGC**TTTTATTATTAGGAGTTGCTTG | *comD* (no stop codon) |
| ComD-SpeI-F  His-BamHI-B | GG**ACTAGT**ATGAATGAAGCCTTAATGATACT  CG**GGATCC**CAAAAAACCCCTCAAGACC | add His-tag |
| S29-T32-F | CG**GGATCC**TACCTTAGAAAATAGAAACAAGAGA | loopA1 |
| S29-T32-B | CG**GGATCC**AAAAAGGAATTAACTCTTTTTTCGA |  |
|  |  |  |
| S29-K36-F  S29-K36-B | CG**GGATCC**TACCTTAGAAAATAGAAACAAGAGA  CG**GGATCC**GAATTAACTCTTTTTTCGATAAGC | loopA2 |
|  |  |  |
| L107-T110-F  L107-T110-B | CG**GGATCC**GATAAAGAATATGATTGCCCG  CG**GGATCC**CAAGGAATTGTAATGGGCAGTAG | loopB1 |
|  |  |  |
| Q111-V114-F  Q111-V114-B | CG**GGATCC**ATGGGCAGTAGCATTATAAC  CG**GGATCC**AGTTCCATCCAAGATAAAG | loopB2 |
|  |  |  |
| N184-P187-F  N184-P187-B | CG**GGATCC**ATAACTCTCTATAACATACAATAC  CG**GGATCC**ACTTTAAAATTTCGTAAATTTGTC | loopC1 |
|  |  |  |
| T188-F191-F  T188-F191-B | CG**GGATCC**CGGTATCACATTATAACTCTC  CG**GGATCC**CGTAAATTTGTCGTTATTGTCT | loopC2 |
|  |  |  |
| NV>AA-F  NV>AA-B | GAGAGTTAT**GCAGCG**ATACCGACTTTAAAATTTCGT  ACGAAATTT**CGCTGC**CGGTATCACATTATAACTCTC | loopC3 |
|  |  |  |
| IP>AA-F  IP>AA-B | GAGAGTTATAATGTG**GCAGCG**ACTTTAAAATTTCGT  ACGAAATTTTAAAGT**CGCTGC**CACATTATAACTCTC | loopC4 |
|  |  |  |
| PCipB-F  PCipB-B | GG**GGTACC**TCATTTTATATCTCCTTTTTTTGATT  AA**CTGCAG**ATGATAAATACCCCTTCCCCA | pCipB-lux reporter |
|  |  |  |
| PnlmAB-F  PnlmAB-B | GG**GGTACC**GTAAGTCGAGGTGCTTTAGCA  AA**CTGCAG**TTGTTCAAATGCCTGTGTATC | pNlmAB-lux reporter |
|  |  |  |
| PnlmD-F  PnlmD-B | GG**GGTACC**AGGATTATAAGTCCAACGAAG  AA**CTGCAG**ATTCATATGATAGATACCTCTTTTC | pNlmD-lux reporter |
|  |  |  |

*: Restriction sites are bolded: GGATCC, BamHI; GGTACC, KpnI; CTCGAG, XhoI; TCTAGA, XbaI; GGCGCGCC, AscI; GGCCGGCC, FseI; GCGGCCGC, NotI; CTGCAG, PstI; GAATTC, EcoRI; GCATGC, SphI, GGTACC, KpnI*.*


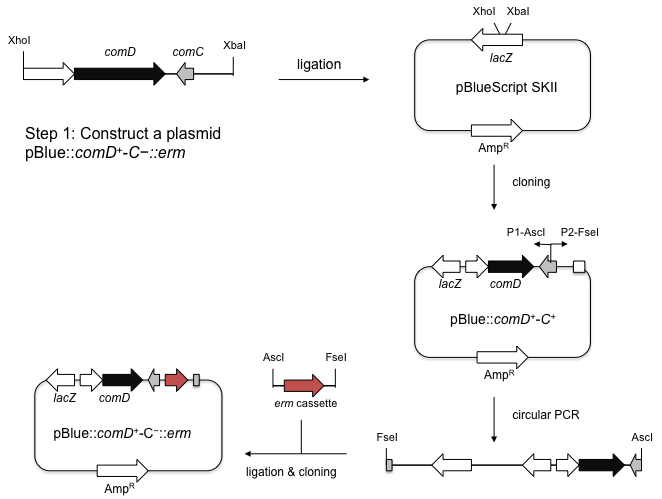


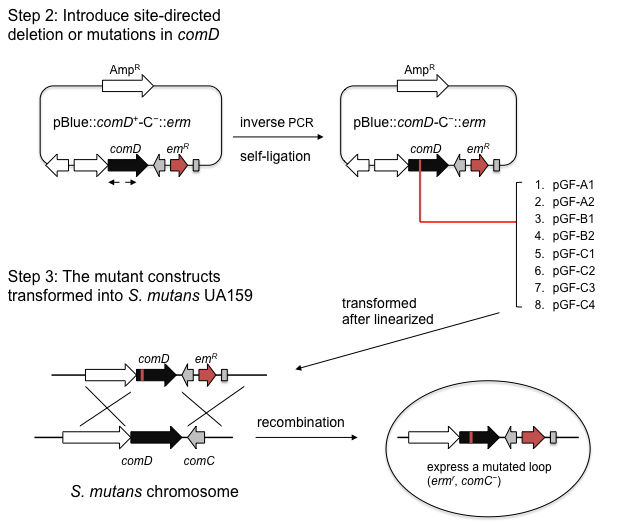


**Fig. S1.** A schematic diagram describes the construction of the loopA, loopB and loopC mutants by a three-step genetic approach. In the first step, a 2064-bp DNA fragment containing the *comD*-coding sequence and its flanking regions was amplified by PCR against the genomic DNA of *S. mutans* UA159, and subcloned into pBlueScript SKII (Stratagene). The resulting plasmid was then used as a template to generate an amplicon (the entire plasmid) with two restriction sites of *Asc*I and *Fse*I by circular PCR from the location downstream of *comD* but within *comC*. The amplicon was digested and ligated to the same restriction sites of an erythromycin resistance cassette. The ligation product was cloned into an *E. coli* host, generating a new plasmid, pGF-C0 that contained a wild copy of *comD* (ComD^+^) but with *comC* (ComC^−^) inactivated by the insertion of the *erm* cassette. In the second step, this new plasmid was used as a template to generate six deletion constructs of loopA, loopB and loopC by an inverse PCR strategy and two alanine substitution mutant constructs corresponding to the target codons of loopC by a QuickChange II site-directed mutagenesis kit. The resulting products were digested with *BamH*1, self-ligated and cloned into *E. coli*, generating new plasmids that harbored a mutant construct of loopA, loopB or loopC, respectively. All the plasmids containing a mutant construct were genetically confirmed by sequencing. In the last step, these plasmids were linearized and transformed into *S. mutant* UA159. Following double-crossover recombination, each of these constructs was integrated into the *S. mutans* genome, generating numerous loopA, loopB and loopC mutants. Positive transformants were selected from THYE plates plus erythromycin (10 μg/ml). Their location and orientation in the genome were confirmed again by a PCR strategy using a combination of four primers specific to *comD* and the *erm* cassette. A *S. mutant* strain with the wild copy *comD* but with *comC* inactivated by the insertion of the *erm* cassette (ComD^+^, ComC^−^, Erm^r^) was included as a positive control, while *comD* deletion mutant (ΔcomD) was used as a negative control.


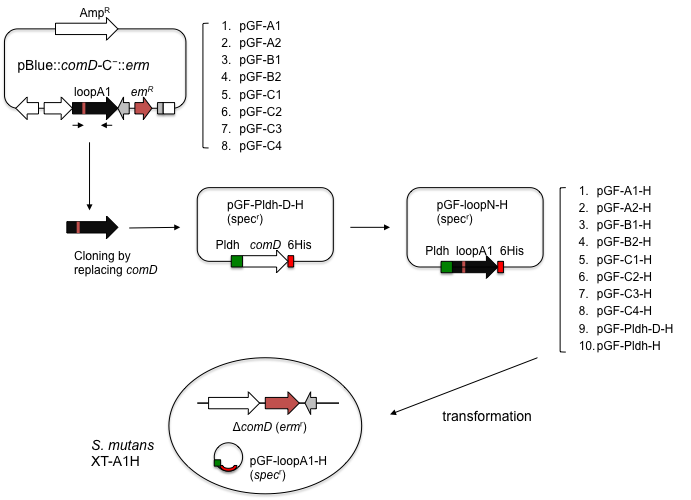


**Fig. S2.** A schematic diagram describes the construction of *S. mutans* strains that constitutively express a His-tagged, ComD mutant protein. The loopA, loopB and loopC constructs (except stop codon) were amplified by PCR using primers ComD-BamHI-F and ComD-NotI-B. The PCR products were digested, purified and cloned into a vector pGF-Pldh-D-H by replacing the *comD*. Each plasmid was constructed in such a way that the mutant *comD* coding sequence representing each mutated loop was fused to the *ldh* promoter (P*_ldh_*) with its start codon and to 6His-tag with its end, generating a number of new plasmids. The new plasmids were confirmed by PCR and sequencing. A plasmid pGF-Pldh-H (without *comD*) was also constructed as a negative control simply by removing the *comD* from pGF-Pldh-D-H. The confirmed plasmids were transformed into the chromosomally deleted *comD* (Δ*comD*) mutant, generating a new set of *S. mutans* strains that constitutively express a His-tagged mutant *comD* protein and facilitating detection of these mutant proteins by Western blotting using the anti-His antibody.


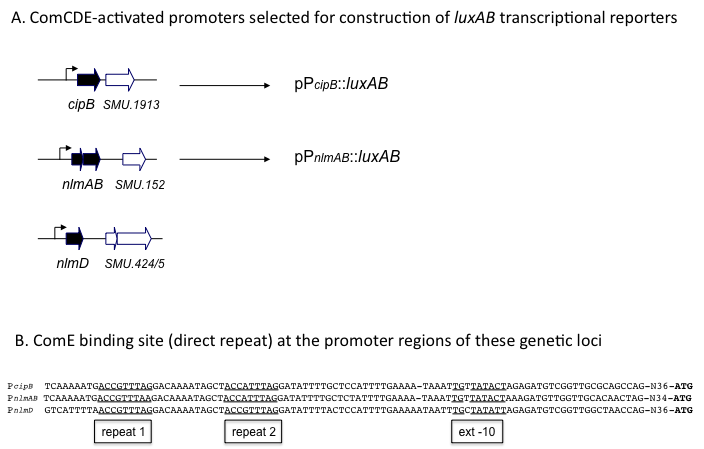


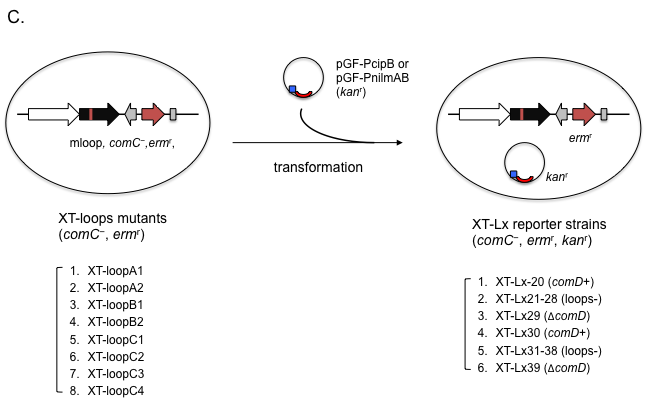


**Fig. S3 A.** A schematic diagram describes selection of two CSP-inducible promoters from three bacteriocin-encoding genes, *cipB* (P*cipB*), *nlmAB* (P*nlmAB*) and *nlmD* (P*nlmD*) to construct *luxAB* transcriptional reporters. **B.** Their promoters contain the consensus ComE binding site (direct repeat) and are directly controlled by the ComCDE quorum sensing system. **C.** The reporter plasmids constructed were transformed into individual *S. mutans* mutants, generating two groups of *luxAB* reporter strains.
